# Supplementary material for: Trends and factors associated with pneumonia mortality at Buea Regional Hospital in Cameroon from 2020 to 2023
Source: PLoS One. 2025 Dec 9;20(12):e0313234. doi: 10.1371/journal.pone.0313234 (PMC12688150; doi:10.1371/journal.pone.0313234)
Supplement: Attachment — Submitted filename: PONE-D-24-47412.docx [file pone.0313234.s003.docx]

General comments:

Distinguishing between typical and atypical pneumonia clinically is difficult; for example, patient may present with dry cough and low-grade fevers (criteria used to define atypical pneumonia) and still have findings on chest x-ray that are characteristic of typical pneumonia. Patients with COVID-19 diagnosis were excluded from the analysis but it is unclear if COVID testing was offered to all patients with suspected pneumonia. It is highly plausible that a significant number of patients with typical/atypical pneumonia diagnosis had COVID. I would recommend combining all the pneumonia cases and redoing analysis.

Need to discuss limitations of the study.

Specific comments:

43…………………………………………………………………..This implies there

44 is need for prompt diagnosis and treatment of Pneumonia cases with amoxicillin

clavulanic acid in order to reduce the case fatality rate in Buea Health District

*There are many appropriate antibiotics for treatment of pneumonia and would not limit the recommendation to amoxicillin-clavulanic.*

97………………………………………………………………………………………..Only patients clinically

98 and radiologically diagnosed with typical or atypical pneumonia (excluding tuberculosis)

99 were considered for this study.

*Can you provide details on criteria used for clinical diagnosis of pneumonia, atypical pneumonia and how pneumonia was differentiated from TB, COVID or other viral pneumonia using clinical diagnosis. Also, it is not clear from the results which patients were diagnosed using clinical criteria vs. radiographic criteria vs. both.*

142…………………Out of these, 1,020 were identified as patients diagnosed with Pneumonia

143 (fig 1). After excluding those with incomplete data and those diagnosed with COVID-19

and Tuberculosis, 746 files were retained for the study.

*I think it would be helpful if you can talk about some challenges using clinical diagnosis. For example, someone diagnosed with pneumonia may end up being diagnosed with TB and differentiating between typical and atypical pneumonia may not be possible using clinical diagnosis alone. Also, in figure 1, 159 patients diagnosed with pneumonia were excluded because they had COVID-19. Was COVID-19 testing offered to all patients with pneumonia? If not, this is a limitation that needs to be addressed as other patients with COVID pneumonia are most likely represented in the 746 participants included in the analysis.*

*Figure one: blurry, please submit a clearer image.*

170……………………………………………More cases of typical pneumonia were reported in

2021 especially during the month of September.

*How do you explain this? Was there a change in how the hospital defined pneumonia? Could this be due to COVID-19?*

216 Logistic regression analysis showed that being hospitalized for less than 10 days and not treated with amoxicillin-clavulanic acid were 10 times and 3 times independently and

significantly associated with a dead outcome respectively for admitted pneumonia

*It seems like the majority 88% (581/662) of participants were treated with antibiotics (256 monotherapy + 406 combined) What other antibiotics were prescribed besides amoxicillin-clavulanic?*

241…. Although we excluded all patients positive for COVID-19 in this study, there is a possibility that a good number of the typical/atypical pneumonia cases diagnosed in 2021 were equally positive for COVID-19 or previously had a recent COVID-19 infection which weakened their immune system and gave way for bacterial pneumonia to set in as a secondary infection.

*COVID-19 in itself can cause pneumonia, please revise. Also, was the policy in BHD to test all patients admitted with pneumonia for COVID-19? If not, it would seem like other patients with COVID-19 pneumonia were included in the analysis.*

252 …………………………We however came across studies [16–18] which also revealed a decreasing trend in pneumonia case fatality over time (but not 2020 to 2023) as seen in our study. This overall decrease in case fatality rate over time could be due to improvements in the diagnosis and management of the disease.

*There’s a notable decrease in trends in pneumonia case fatality even though the total number of patients with pneumonia remained the same. Are you able to provide additional details on what the hospital was/is doing to improve diagnosis and management?*

258 Majority (72.5%) of the pneumonia patients who died in our study were above 50 years of age and this could be attributed to weakened immune systems thus necessitating prompt diagnosis and early treatment for a favorable outcome.

*Do you have any data on the types and frequency of pathogens isolated from patients with pneumonia? For those pathogens with available vaccines (e.g., pneumococcal pneumonia, H. flu, COVID, influenza), vaccination may offer better protection and overall reduction in sickness and death.*

275…………………In fact, combining clavulanic acid to amoxicillin does not come with any added advantage (neither does it come with a disadvantage) for the elimination of *Streptococcus pneumoniae*[33]*.*

*This is not accurate. Some patients could have been infected with resistant bacteria that will respond to amoxicillin-clavulanic over amoxicillin alone. Unless you have drug sensitivity data on all pneumonia isolate, I would not say that Amox-clav does not add any advantage. Please revise.*
