# Supplementary material for: Trends and factors associated with pneumonia mortality at Buea Regional Hospital in Cameroon from 2020 to 2023
Source: PLoS One. 2025 Dec 9;20(12):e0313234. doi: 10.1371/journal.pone.0313234 (PMC12688150; doi:10.1371/journal.pone.0313234)
Supplement: Attachment — Submitted filename: RESPONSE TO REVIEWERS.docx [file pone.0313234.s006.docx]

**RESPONSE TO REVIEWERS**

Journal requirements:

1.Please ensure that your manuscript meets PLOS ONE's style requirements, including those for file naming. The PLOS ONE style templates can be found at

https://journals.plos.org/plosone/s/file?id=wjVg/PLOSOne_formatting_sample_main_body.pdf and

[https://journals.plos.org/plosone/s/file?id=ba62/PLOSOne_formatting_sample_title_authors_affiliations.pdf](https://track.editorialmanager.com/CL0/https:%2F%2Fjournals.plos.org%2Fplosone%2Fs%2Ffile%3Fid=ba62%2FPLOSOne_formatting_sample_title_authors_affiliations.pdf/1/010f0198798e173b-e48bade7-2cdc-4ede-9bc6-f946b5580452-000000/v1spuLWQTx7yzTJ11BsWMUId0yJ7SVb44n7ppJpaUOo=222)

RESPONSE

Manuscript has been revised to meet PLOS ONE's style requirements

Additional Editor Comments (if provided):
1. Thank you for submissing your manuscript to PLOSONE. There are some merit in the work, however, there are significant issue in the write-up. There is need so check grammar across the manuscript and also make sure to italize where necessary.

RESPONSE

We have checked and corrected grammatic errors to the best of our knowledge

2. There is need to expand on the inclusion and exclusion for the study

RESPONSE:

More detailed inclusion and exclusion criteria not stated in the methods (line 101-110)

**REVIEWER #1:**

General comments:

Distinguishing between typical and atypical pneumonia clinically is difficult; for example, patient may present with dry cough and low-grade fevers (criteria used to define atypical pneumonia) and still have findings on chest x-ray that are characteristic of typical pneumonia. Patients with COVID-19 diagnosis were excluded from the analysis but it is unclear if COVID testing was offered to all patients with suspected pneumonia. It is highly plausible that a significant number of patients with typical/atypical pneumonia diagnosis had COVID. I would recommend combining all the pneumonia cases and redoing analysis.

Need to discuss limitations of the study.

RESPONSE:

Thank you for your valuable suggestion. We have now grouped atypical and typical pneumonia. Its just referred to as “pneumonia” at this point.

We actually highlighted the limitations of the study in the different paragraphs (where applicable) at the discussion section. May be that wasn’t clear enough. We have now dedicated a paragraph for limitations of the study in the discussion section (line 300-316)

Specific comments:

43…………………………………………………………………..This implies there

44 is need for prompt diagnosis and treatment of Pneumonia cases with amoxicillin

clavulanic acid in order to reduce the case fatality rate in Buea Health District

*There are many appropriate antibiotics for treatment of pneumonia and would not limit the recommendation to amoxicillin-clavulanic.*

RESPONSE:

Thank you for your remark. The “Guide to antibiotic therapy for common bacterial diseases in Cameroon” (released in 2024) recommends the use of amoxicillin-clavulanic-acid (as well as other antibiotics) for the treatment of pneumonia. We capitalized on amoxicillin-clavulanic-acid because according to the above-mentioned guide, all the different forms of bacterial pneumonia have amoxicillin-clavulanic-acid as a treatment option (either first choice or second choice). Our study actually shows that if amoxicillin-clavulanic-acid is included in the treatment, there is a higher chance of avoiding dead as a result of the pneumonia. In summary, the recommendation we made in our study is strictly based on our findings and IT DOES NOT BY ANY CHANCE imply that only amoxicillin-clavulanic-acid should be used for treating pneumonia. We have however rephrased the sentence (line 42 to 45)

97………………………………………………………………………………………..Only patients clinically

98 and radiologically diagnosed with typical or atypical pneumonia (excluding tuberculosis)

99 were considered for this study.

*Can you provide details on criteria used for clinical diagnosis of pneumonia, atypical pneumonia and how pneumonia was differentiated from TB, COVID or other viral pneumonia using clinical diagnosis. Also, it is not clear from the results which patients were diagnosed using clinical criteria vs. radiographic criteria vs. both.*

RESPONSE

Thank you for your comment. We have now included in the methods a subheading describing how diagnosis pneumonia, COVID-19 and Tuberculosis were considered (line 111-123)

142…………………Out of these, 1,020 were identified as patients diagnosed with Pneumonia

143 (fig 1). After excluding those with incomplete data and those diagnosed with COVID-19

and Tuberculosis, 746 files were retained for the study.

*I think it would be helpful if you can talk about some challenges using clinical diagnosis. For example, someone diagnosed with pneumonia may end up being diagnosed with TB and differentiating between typical and atypical pneumonia may not be possible using clinical diagnosis alone. Also, in figure 1, 159 patients diagnosed with pneumonia were excluded because they had COVID-19. Was COVID-19 testing offered to all patients with pneumonia? If not, this is a limitation that needs to be addressed as other patients with COVID pneumonia are most likely represented in the 746 participants included in the analysis.*

RESPONSE:

Thank you for pointing this out. This has been included as a limitation to the study in the designated paragraph under discussion section. (Line 309-316)

*Figure one: blurry, please submit a clearer image.*

RESPONSE

A clearer image has now been submitted

170……………………………………………More cases of typical pneumonia were reported in

2021 especially during the month of September.

*How do you explain this? Was there a change in how the hospital defined pneumonia? Could this be due to COVID-19?*

RESPONSE:

This was most likely due to COVID-19. Check the first paragraph of the discussion which also stands as a limitation of the study

216 Logistic regression analysis showed that being hospitalized for less than 10 days and not treated with amoxicillin-clavulanic acid were 10 times and 3 times independently and

significantly associated with a dead outcome respectively for admitted pneumonia

*It seems like the majority 88% (581/662) of participants were treated with antibiotics (256 monotherapy + 406 combined) What other antibiotics were prescribed besides amoxicillin-clavulanic?*

RESPONSE

Other antibiotics like Azithromycin, metronidazole, amoxicillin etc were equally prescribed. However, we focused on amoxicillin-clavulanic-acid because according to The “Guide to antibiotic therapy for common bacterial diseases in Cameroon”, all the different forms of bacterial pneumonia have amoxicillin-clavulanic-acid as a treatment option (either first choice or second choice) so we wanted to really see how useful or relevant amoxicillin-clavulanic-acid is in this context.

241…. Although we excluded all patients positive for COVID-19 in this study, there is a possibility that a good number of the typical/atypical pneumonia cases diagnosed in 2021 were equally positive for COVID-19 or previously had a recent COVID-19 infection which weakened their immune system and gave way for bacterial pneumonia to set in as a secondary infection.

*COVID-19 in itself can cause pneumonia, please revise. Also, was the policy in BHD to test all patients admitted with pneumonia for COVID-19? If not, it would seem like other patients with COVID-19 pneumonia were included in the analysis.*

RESPONSE

We know that COVID-19 causes pneumonia. However, it was not part of our interest. During the said period, all clinically suspected pneumonia cases were tested for COVID-19. Unfortunately, we can’t say with certainty that we successfully excluded all COVID-19 cases from the study so this just has to come up as one of the limitations of the study. Check the paragraph addressing limitations in the discussion section.

252 …………………………We however came across studies [16–18] which also revealed a decreasing trend in pneumonia case fatality over time (but not 2020 to 2023) as seen in our study. This overall decrease in case fatality rate over time could be due to improvements in the diagnosis and management of the disease.

*There’s a notable decrease in trends in pneumonia case fatality even though the total number of patients with pneumonia remained the same. Are you able to provide additional details on what the hospital was/is doing to improve diagnosis and management?*

RESPONSE

Thank you for pointing this out. There is a possibility that the COVID-19 pandemic led to better triage systems and early recognition of respiratory distress, which also benefited non-COVID pneumonia patients who came to the hospital. The pandemic may have also brough in more emphasis on pulse oximetry and rapid initiation of oxygen in hypoxemic patients. The presence of a pulmonologist at the hospital may also have contributed to better case detection and management, leading to improved survival over time.

This has been added in the discussion section of the manuscript (line 258-263)

258 Majority (72.5%) of the pneumonia patients who died in our study were above 50 years of age and this could be attributed to weakened immune systems thus necessitating prompt diagnosis and early treatment for a favorable outcome.

*Do you have any data on the types and frequency of pathogens isolated from patients with pneumonia? For those pathogens with available vaccines (e.g., pneumococcal pneumonia, H. flu, COVID, influenza), vaccination may offer better protection and overall reduction in sickness and death.*

RESPONSE

Thank you for pointing this out. This information is lacking. Check the paragraph addressing limitations in the discussion section.

275…………………In fact, combining clavulanic acid to amoxicillin does not come with any added advantage (neither does it come with a disadvantage) for the elimination of *Streptococcus pneumoniae*[33]*.*

*This is not accurate. Some patients could have been infected with resistant bacteria that will respond to amoxicillin-clavulanic over amoxicillin alone. Unless you have drug sensitivity data on all pneumonia isolate, I would not say that Amox-clav does not add any advantage. Please revise.*

RESPONSE

The statement you mentioned above is accurate as long as the organism in question is *Streptococcus pneumoniae.* We talked about this lack of advantage of clavulanic acid addition strictly for *Streptococcus pneumoniae.* Amoxicillin Resistance in S. pneumoniae is **not** due to β-lactamase production. Instead, resistance arises from **altered PBPs** (target modification). Since clavulanic acid only blocks β-lactamase enzymes and S. pneumoniae does not produce them, **adding clavulanic acid does not enhance amoxicillin’s activity** against S. pneumoniae. However, as clearly written in the manuscript, there is added advantage when we start considering other bacterial pathogens that may cause pneumonia that is why the use of Amox-clav combination is still a better approach. Please read the paragraph in question till the end. However, since you seem not to be comfortable with that particular sentence, the sentence has been deleted in the revised manuscript. Thank you

**REVIEWER #2:**

This is a well-executed, relevant, and timely retrospective study assessing the trends and predictors of pneumonia mortality in a low-resource setting. It addresses a crucial public health concern and fills a significant gap in the regional literature, particularly from Sub-Saharan Africa. The methodology is robust, and the findings are valuable for healthcare planning and clinical practice. However, there is some room for improvement.

Here are my comments:

1. Introduction

Rephrase verbose or redundant parts, e.g., "Pneumonia cases like wise deaths..." to "Pneumonia cases and associated deaths..."

Use more precise language: "It proves to us..." to "It suggests..." or "It indicates..."

Avoid generalizations without sources, e.g., “...helps in predicting future outbreaks...” (requires clarification or reference).

RESPONSE

Thank you for pointing these out all the corrections have been made. As for the “...helps in predicting future outbreaks...”, that statement is just an obvious fact in epidemiology and can apply for any disease as long as it shows a periodic pattern in its occurrence over time. If we see a pattern in the occurrence of a disease over the years e.g the number of cases always rise above expected in the month of March, we can comfortably predict that the disease would have an epidemic or an outbreak in the upcoming month of March 2026. We might be wrong but we don’t think that needs a reference. It’s an obvious logic.

2. Methods

Clarify diagnostic criteria for classifying typical vs. atypical pneumonia—were these physician-noted, ICD-coded, or based on lab/radiology?

RESPONSE

Thank you for pointing this out. A subheading (Diagnosis of pneumonia) has been added in the methods section to address this (line 111-123)


It’s unclear how pneumonia types were classified in outpatient cases without radiological confirmation.

RESPONSE

Diagnosis of pneumonia in this study was considered only when both clinical and radiological findings were available to confirm it. Every out-patient case without radiological confirmation records were considered as patient with missing/incomplete data and so was not included in the 746 cases (listwise deletion)

Mention how missing or incomplete records were handled statistically (listwise deletion or imputation?).

RESPONSE

Thank you for pointing this out. We handled missing or incomplete records by listwise deletion (line 149-151)

3. Results

Provide numbers alongside percentages throughout. For instance, instead of only “13.7% fatality rate,” also state “91 out of 662.”

RESPONSE

Numbers have now been provided alongside percentages were applicable

If possible, distinguish between univariate and multivariate analysis outcomes.

RESPONSE

All the relevant details is seen in table 2 (crude odds ratio is the univariate analysis while adjusted odds ratio is the multivariate analysis. Only variables with **p-value ≤ 0.05** in the bivariate analysis were considered eligible for inclusion in the multivariate regression model

4. Discussion

Avoid speculative language like "proves to us". Replace with "suggests" or "indicates."

RESPONSE

Speculative languages have been removed

Clarify limitations explicitly, e.g.:

A.Retrospective design limits causality.
B. Lack of pathogen confirmation (no lab cultures).
C. Possible misclassification bias due to reliance on clinical diagnoses.

Consider adding a brief note on potential interventions (e.g., increased community awareness, pneumococcal vaccination, antibiotic stewardship).

RESPONSE

An entire paragraph in the discussion section have been dedicated for limitations (line 300-316)

5. Conclusion

Consider including a final sentence emphasizing policy or clinical recommendations for Buea Health District or similar contexts.

RESPONSE

To the best of our knowledge, the last sentence in the conclusion paragraph addresses this need.

**REVIEWER #3:**

Dear author(s),

Congratulations on your study examining mortality trends and associated factors in patients with typical and atypical pneumonia in Cameroon. I have read your study carefully. Please find my suggestions and questions below.

Is there a specific reason for examining harvests from 2020 to 2023?

RESPONSE

Thank you for this question. Hospital records from 2020 onward were **readily available, and accessible** for review. In addition, this period overlaps with the **COVID-19 pandemic**, which had a profound impact on pneumonia diagnosis, hospital attendance, and respiratory disease burden. A **4-year span** provides a reasonable time frame to observe **trends, fluctuations, and seasonal variations,** while keeping the dataset manageable and relevant

Line 62: ‘Generally, pneumonia is usually classified as typical or atypical’ should be changed to ‘Pneumonia is classified as typical or atypical based on its clinical features.’

RESPONSE

Statement has been changed

You provided the rationale in the last paragraph of the introduction. You should write the purpose of your study at the end.

RESPONSE

The last sentence in the introduction section now carries the purpose/aim of the research (line 85-87)

In the materials and methods section, you should describe the clinical characteristics of typical and atypical pneumonia (provide definitions). When providing these definitions, it would be helpful to mention the guidelines used by doctors in your country for the diagnosis and treatment of pneumonia.

RESPONSE

We have just grouped all as pneumonia following a recommendation from the first reviewer. We have also added a new sub heading in the methods section addressing how pneumonia diagnosis was considered

You found that mortality increases in patients with a hospital stay of less than 10 days. Why did you set the hospital stay at 10 days? Do you have any references for this?

RESPONSE

The 10-day cut-off for hospital stay was selected because it approximated the median length of stay observed in our dataset, and it also aligns with previous studies showing that the majority of pneumonia-related deaths occur within the first 7–10 days of admission (Waterer GW, et al. In-hospital deaths among adults with community-acquired pneumonia: early fatalities cluster in the first 10 days. Chest (or journal title) 2018.

Montull B, et al. Predictors of severe sepsis and associated in-hospital mortality: median length of stay and risk of early death. Crit Care (or journal title) 2016)

Using this threshold allowed us to distinguish between patients who experienced early in-hospital mortality, often reflecting more severe disease, and those who survived longer, entering a recovery phase.”

A sentence has been added in the discussion section to address this (line 270-272)

The first paragraph of the Discussion section should summarise the major results of your study. Then, you should discuss each finding in a separate paragraph.

RESPONSE

Thank you for this feedback. We however addressed the different results briefly in the designated paragraphs carrying their respective discussions and so we feel like adding a separate paragraph again just to summarize major results would be repetitive.
Sincerely.
