# Supplementary material for: Trends and factors associated with pneumonia mortality at Buea Regional Hospital in Cameroon from 2020 to 2023
Source: PLoS One. 2025 Dec 9;20(12):e0313234. doi: 10.1371/journal.pone.0313234 (PMC12688150; doi:10.1371/journal.pone.0313234)
Supplement: Attachment — Submitted filename: Response_to_reviewers_auresp_2.docx [file pone.0313234.s007.docx]

**RESPONSE TO REVIWERS**

JOURNAL REQUIREMENTS:

If the reviewer comments include a recommendation to cite specific previously published works, please review and evaluate these publications to determine whether they are relevant and should be cited. There is no requirement to cite these works unless the editor has indicated otherwise.

RESPONSE:

Recommendation to cite specific previously published works was reviewed and deemed relevant

2. Please review your reference list to ensure that it is complete and correct. If you have cited papers that have been retracted, please include the rationale for doing so in the manuscript text, or remove these references and replace them with relevant current references. Any changes to the reference list should be mentioned in the rebuttal letter that accompanies your revised manuscript. If you need to cite a retracted article, indicate the article’s retracted status in the References list and also include a citation and full reference for the retraction notice.

RESPONSE:

We have reviewed our reference list and to he best of our knowledge, none of the references have been retracted. However, if you think otherwise, please indicate to us the particular retracted reference so that we can do the needful.

REVIEWER

This is a much better version of the manuscript than the first one, but additional revisions are still needed. Below are some recommendations that can strengthen the paper. Consider a review by someone with experience in technical editing before resubmission.

RESPONSE

Thank you for your dedication in reading and correcting this work.

29 Only patients clinically and radiologically diagnosed with pneumonia of bacterial origin (excluding tuberculosis) were considered for this study.

*Consider revising to “**Only patients clinically and radiologically diagnosed with pneumonia (excluding TB and COID-19) were considered for this study”. Other viruses can cause pneumonia and since you only tested for TB and COVID, it is best to keep this broad and not limit to pneumonia with bacterial origin*.

RESPONSE

Correction done as requested. (line 29 -30)

35 …. There was a decreasing trend in case fatality rate across the years with a general case fatality rate of 13.7%.

*Need to add the case fatality rate by year to show the trend.*

RESPONSE

Correction done as requested. (line 35 -36)

39… There was a fluctuating trend in pneumonia cases in Buea regional hospital from 2020 to 2023 implying that pneumonia is endemic in Buea Health district

*I believe the intend is to highlight the year-round (as opposed to seasonal) high rates of pneumonia infection in the district. The above sentence does not bring this out, please revise*

RESPONSE

Correction done as requested. (line 40 -42)

61………………… …………………………………..It can be caused by several bacteria, viruses and fungi [7]. Generally, pneumonia is classified as typical or atypical based on its clinical features or causative agent. Typical pneumonia refers to a more severe form of pneumonia usually caused by *Streptococcus pneumoniae* (majority of the cases), *Staphylococcus aureus, Haemophilus influenzae* and other Gram-negative bacteria that can survive in the presence of oxygen [8].

*I would recommend removing the distinction between typical and atypical pneumonia and just focusing on pneumonia as diagnosed by clinical and radiographic criteria. You’ve already done so in the result section (for the most part). The distinction does not help in addressing the main objectives of the paper (identifying the trend of pneumonia and factors associated with pneumonia mortality at the Buea regional hospital from 2020 to 2023). You can still include the leading causes of pneumonia that you’ve highlighted above.*

RESPONSE

Correction done as requested. (line 64 -66)

160.. using line graphs, while description of the various types of pneumonia across the years

*As stated above, the distinction between typical and atypical pneumonia does not add clarity but brings with it confusion given lack of empirical evidence that can allow you to make the distinction.*

RESPONSE

Correction done as requested. (line 156 -157)

173 Figure 1: Review and selection of patient files clinically/radiologically diagnosed with…

Figure 1 is not included. Again, would recommend changing the title to “…patient files clinically/radiologically diagnosed with ~~typical and atypical~~ pneumonia..” similar to the title of Table 1.

RESPONSE

Correction done as requested. (line 168 -169)

235 This shows that pneumonia (especially that caused by bacteria) is endemic throughout the year (not a seasonal disease) in BHD corroborating the findings of other studies

*You should also include right in the beginning of discussion a summary of the other key findings of the study (**relatively high mortality rate that seem to decrease between 2020 and 2023).*

RESPONSE

Correction done as requested. (line 234 -235)

.

246 …were equally positive for COVID-19 or previously had a recent COVID-19 infection which weakened their immune system and gave way for bacterial pneumonia to set in as a secondary infection…

*Please add a reference about recent COVID infection weakening patients immune system*.

RESPONSE

Reference has added as requested. (line 248)

265 ……. Majority (72.5%) of the pneumonia patients who died in our study were above 50 years of age and this could be attributed to weakened immune systems.

*Recommend changing “weakened immune systems” to* *presence of other co-morbidities. This is broad and not as specific as weekend immune system*.

RESPONSE

Correction done as requested. (line 268)

284… stand-alone amoxicillin (without clavulanic acid) is usually good enough for eliminating *Streptococcus pneumoniae*

*Recommend changing “good enough for eliminating” to something like* *appropriate for treating Streptococcus… Elimination can mean different things clinically and microbiologically.*

RESPONSE

Correction done as requested. (line 285 -286)

297….. there is a higher chance of avoiding dead as a result of the pneumonia

*Consider revising to something like* *there is reduced likelihood of death compared to when this antibiotic is not used.*

RESPONSE

Correction done as requested. (line 297 -299)

312……………… As such, its quite difficult to distinguish between typical and atypical pneumonia

*No need to include this, please delete.*

RESPONSE

Correction done as requested.
